# The Eastern Fox Squirrel (*Sciurus niger*) exhibits minimal patterns of phylogeography across native and introduced sites

**DOI:** 10.1093/jmammal/gyae133

**Published:** 2024-11-15

**Authors:** Noah Armstrong, Dylan M Klure, Robert Greenhalgh, Tess E Stapleton, M Denise Dearing

**Affiliations:** School of Biological Sciences, University of Utah, 257 S. 1400 E. Room 201, Salt Lake City, UT 84102, United States; School of Biological Sciences, University of Utah, 257 S. 1400 E. Room 201, Salt Lake City, UT 84102, United States; School of Biological Sciences, University of Utah, 257 S. 1400 E. Room 201, Salt Lake City, UT 84102, United States; School of Biological Sciences, University of Utah, 257 S. 1400 E. Room 201, Salt Lake City, UT 84102, United States; ARUP Laboratories, 500 Chipeta Way, Salt Lake City, UT 84108, United States; School of Biological Sciences, University of Utah, 257 S. 1400 E. Room 201, Salt Lake City, UT 84102, United States

**Keywords:** fox squirrel, introduced species, population genetics, rodent, whole genome sequencing, ardilla zorro oriental, especies introducidas, genética de poblaciones, roedor, secuenciación del genoma

## Abstract

Introduced species are one of the leading causes of declining global biodiversity and result in many billions of dollars of losses to the bioeconomy worldwide. Introduced species have become increasingly common due to globalization and climate change, and population genetics is a useful tool for the management of such species. The Eastern Fox Squirrel (*Sciurus niger*) is a highly successful invader that was introduced to many states in western North America throughout the 20th century. We used low-pass whole genome sequencing to evaluate phylogeographic structure across native and introduced ranges of this species and identify the putative number and geographic sources of introductions in California and Utah. We found minimal patterns of phylogeographic structure, consistent with recent range and population expansion since the Last Glacial Maximum. Additionally, we found evidence for multiple mitochondrial haplotypes in California and only 1 haplotype in Utah, which suggests that fox squirrels in California were sourced from multiple introduction events while those in Utah were likely sourced from a single event. Genomic resources generated in this study will be useful for future conservation efforts in this species and will assist with the ongoing management of its introductions across western North America.

## Teaser

In the United States, Eastern Fox Squirrel populations are highly genetically similar across its native and introduced ranges. This pattern indicates that this species experienced rapid population growth and range expansion since the end of the last ice age (~12,000 years ago).

## Resumen

Especies introducidas son una de las principales causes de la disminución mundial de la biodiversidad y resultan en pérdidas de miles de millones de dólares para la bioeconomía mundial. La genética de poblaciones es un instrumento útil para el manejo de estas especies, cada vez más comunes debido a la globalización y el cambio climático. La ardilla zorro oriental (*Sciurus niger*) es una especie invasora exitosa que se introdujo en muchos estados del oeste de América del Norte a lo largo del siglo XX. Utilizamos secuenciación de baja cobertura del genoma para evaluar la estructura filogeográfica de la ardilla zorro oriental a través sitios nativos e introducidos, para identificar el número de introducciones en los estados de California y Utah, y para determinar orígenes geográficos de estas introducciones. Encontramos una señal mínima de estructura filogeográfica, consistente con la reciente expansión de rango y población de esta especie desde el ultimo Máximo Glacial. Además, encontramos múltiples haplotipos mitocondriales en California y solo uno haplotipo en Utah. Esto sugiere que en California, la ardilla zorro oriental se originó de múltiples eventos de introducción, mientras en Utah se originó a partir de un solo evento. Los recursos genómicos de este estudio serán útiles para futuros proyectos de conservación de esta especie y ayudarán con la gestión de sus introducciones en el oeste de América del Norte.

Invasive species are one of the leading causes of declining global biodiversity and have resulted in the expenditure of an estimated $1.288 trillion USD from 1970 to 2017 (Pyšek et al. 2020; Diagne et al. 2021). Although humans have been facilitating the introduction of flora and fauna into novel environments for over a millennium, the number of introduction events has continued to increase markedly since the industrial revolution. This increase in events is closely tied with increased trade and transport due to globalization (Hulme 2009; Muirhead et al. 2015; Sardain et al. 2019). In addition to these artificial introductions, global climate change may further facilitate the spread of introduced species by altering transportation corridors, climatic conditions, habitat structure, and distributions of native and introduced species, and by changing the effectiveness of existing management strategies (Hellmann et al. 2008; Chown et al. 2015). Management of introduced species is a highly effective strategy with positive conservation outcomes for native species (Langhammer et al. 2024). Biological insight into invasive species is necessary for the development of robust control strategies (McGaughran et al. 2024).

**Fig. 1.**
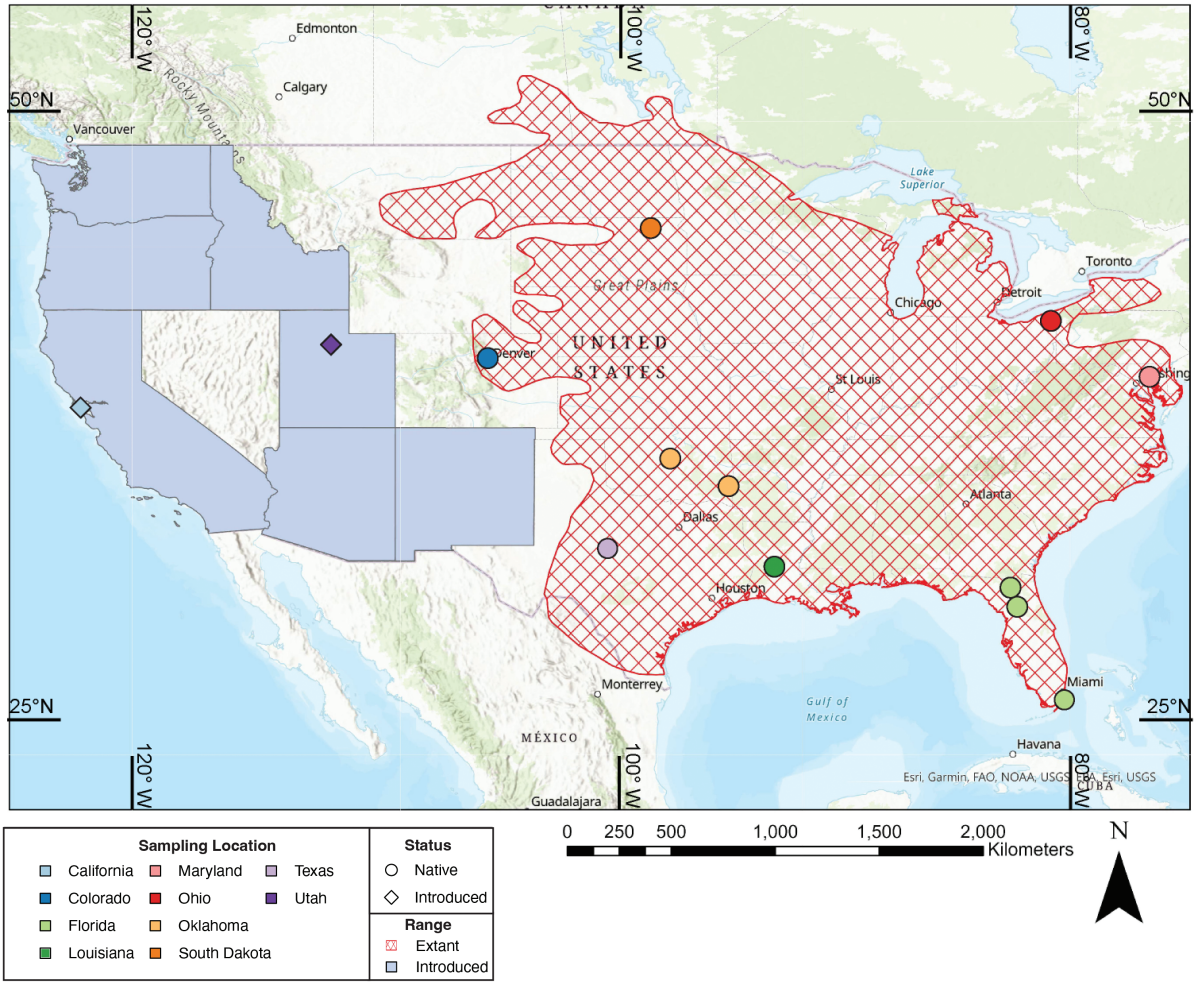
Map showing both native and introduced ranges of *Sciurus niger* in North America and sampling localities for our data set. Areas with hashed lines represent the native range, while bolded states indicate states containing introduced populations. Colors represent the state samples were collected from, and shape represents whether individuals at that locality are introduced or native.

One highly successful introduced species in North America is the Eastern Fox Squirrel (*Sciurus niger*). This species has a large native range encompassing much of the eastern and central United States and has been introduced to many western states including California, Idaho, New Mexico, North Dakota, Oregon, and Washington, and even to Ontario in Canada (Koprowski 1994; Fig. 1). Historical records show that many of these introductions, such as in California, were likely human-mediated and intentional (Claytor et al. 2015). Most recently, fox squirrels were introduced to the state of Utah and were first sighted in the Salt Lake Valley by scientists at the Natural History Museum of Utah in 2011 (Rickart EA, Natural History Museum of Utah, Salt Lake City, Utah, USA, personal communication, 15 December 2023; NHMU 2024). Since then, their population has rapidly increased, and they are now the dominant squirrel species in urban areas. The geographical origin of Utah’s introduced fox squirrel population is currently unknown, although it has been speculated to have been introduced from the western portion of its native range based on similarities in body size and pelage color (Koprowski 1994; Moncrief et al. 2010).

Over the past few decades, population genetics has become a useful tool for the management of introduced species by enabling quantification of their genetic structure, locating geographic sources of introductions, and identifying invasion pathways (Croucher et al. 2013; Browett et al. 2020; North et al. 2021). Historically, partial gene sequences and microsatellite loci have been used to analyze genetic structure in introduced populations (Sunnucks 2000; Yeap et al. 2011; Abdul-Muneer 2014; Sato et al. 2023). Partial sequencing of multiple mitochondrial genes has previously been conducted in *S. niger* in an attempt to characterize phylogeographic structure across its native range, resolve population structure in introduced California populations, and identify the geographic source of California’s introduced populations (Moncrief et al. 2010, 2012; Claytor et al. 2015). However, these efforts provided minimal resolution of the phylogeographic structure of this species across native and introduced sites secondary to minimal genetic differentiation across sampling localities, which was likely further compounded by the limited resolution provided by partial gene sequencing.

Continuous advancements in next-generation sequencing technologies have made possible the use of low-pass whole genome sequencing (WGS) as an economical and high-throughput method for characterizing the genetic structure of introduced and native populations (Chown et al. 2015; Fuentes-Pardo and Ruzzante 2017; Satam et al. 2023). This approach can identify tens of thousands to millions of genome-wide nucleotide polymorphisms to enable a more accurate characterization of genetic structure, potentially even in the face of minimal differentiation. However, a constraint of this approach is that low-pass WGS relies on the availability or curation of high-quality reference assemblies for reference-guided read mapping. Fortunately, the recent development of a reference genome assembly has made possible the use of this technique for the study of the fox squirrel (Kang et al. 2021). The aims of this study were to leverage WGS technologies to resolve the phylogeography of fox squirrels across both their native and introduced ranges and identify the geographic sources and putative number of introduction events in the western states of California and Utah. In addition, we investigated how species distributions of native and introduced tree squirrels have changed in California and Utah over time using publicly available iNaturalist data and we generated genomic resources to aid in the ongoing management of this successful invader.

## Materials and methods.

### Sample collection.

We obtained liver tissue samples for 32 *S. niger* individuals collected from 16 localities in California, Colorado, Florida, Louisiana, Maryland, Ohio, Oklahoma, South Dakota, Texas, and Utah from 1997 to 2023 (Fig. 1; Table 1). South Dakota samples (SD_01, SD_02, and SD_03) were donated by Dr. Eric Pulis (Northern State University), and 1 Utah sample (UT_01) was donated by Dr. Margaret Doolin (University of Utah). All other samples were obtained from museum tissue collections (Table 1). Samples were either stored at −80 °C or at room temperature in 95% EtOH prior to genomic DNA extraction. Although we did not initially intend to include California in this project, we had sufficient resources to add 3 samples to our sequencing lane while achieving our target depth. We selected 3 from a population in California in order to include samples from another introduced population.

**Table 1.** Sample information for all *Sciurus niger* individuals in our data set.

Sample ID:	Museum voucher ID:	Museum	State	County/Parish	Status	Donor
CA_01	MVZ:Mamm:202768	MVZ	CA	Alameda	Introduced	Museum
CA_02	MVZ:Mamm:225663	MVZ	CA	Contra Costa	Introduced	Museum
CA_03	MVZ:Mamm:230668	MVZ	CA	Napa	Introduced	Museum
CO_01	UCM:Mamm:23116	UCM	CO	Jefferson	Native	Museum
CO_02	UCM:Mamm:23121	UCM	CO	Boulder	Native	Museum
CO_03	UCM:Mamm:23165	UCM	CO	Boulder	Native	Museum
FL_01	FLMNH:Mamm:34998	FLMNH	FL	Suwanee	Native	Museum
FL_02	FLMNH:Mamm:34369	FLMNH	FL	Marion	Native	Museum
FL_03	FLMNH:Mamm:34372	FLMNH	FL	Miami-Dade	Native	Museum
LA_01	ASNHC:Mamm:18950	ASNHC	LA	Rapides	Native	Museum
LA_02	ASNHC:Mamm:18914	ASNHC	LA	Rapides	Native	Museum
LA_03	ASNHC:Mamm:19556	ASNHC	LA	Rapides	Native	Museum
MD_01	USNM:Mamm:570207	USNM	MD	Montgomery	Native	Museum
MD_02	USNM:Mamm:569078	USNM	MD	Montgomery	Native	Museum
MD_03	USNM:Mamm:569077	USNM	MD	Montgomery	Native	Museum
OH_01	ASNHC:Mamm:10458	ASNHC	OH	Ashtabula	Native	Museum
OH_02	ASNHC:Mamm:10459	ASNHC	OH	Ashtabula	Native	Museum
OK_01	OMNH: 8754	SNOMNH	OK	Le Flore	Native	Museum
OK_02	OMNH:13968	SNOMNH	OK	Cleveland	Native	Museum
OK_03	OMNH: 12050	SNOMNH	OK	Cleveland	Native	Museum
SD_01	SD_SN_71	N/A	SD	Brown	Native	Collaborator
SD_02	SD_SN_72	N/A	SD	Brown	Native	Collaborator
SD_03	SD_SN_73	N/A	SD	Brown	Native	Collaborator
TX_01	ASNHC:Mamm:16355	ASNHC	TX	Tom Greene	Native	Museum
TX_02	ASNHC:Mamm:20447	ASNHC	TX	Tom Greene	Native	Museum
TX_03	ASNHC:Mamm:10505	ASNHC	TX	Tom Greene	Native	Museum
UT_01	UT_SN_52	N/A	UT	Salt Lake	Introduced	Collaborator
UT_02	UMNH:Mamm:45474	NHMU	UT	Salt Lake	Introduced	Museum
UT_03	UMNH:Mamm:45475	NHMU	UT	Salt Lake	Introduced	Museum
UT_04	UMNH:Mamm:45477	NHMU	UT	Salt Lake	Introduced	Museum
UT_05	UMNH:Mamm:45476	NHMU	UT	Salt Lake	Introduced	Museum
UT_06	UMNH:Mamm:45478	NHMU	UT	Salt Lake	Introduced	Museum

### Library preparation and sequencing.

Total genomic DNA was extracted using a Zymo Research Quick-DNA miniprep Plus Kit (Cat. No. D4068) and following the manufacturer’s protocol. Samples stored in 95% EtOH were washed with 1× phosphate-buffered saline prior to DNA extraction. Following extraction, genomic DNA concentration was measured using an Invitrogen Qubit 4 Fluorometer (Cat. No. Q33238) and an Invitrogen Qubit dsDNA high sensitivity kit (Cat. No. Q32854) following manufacturer’s protocols. All genomic DNA samples were standardized to a concentration of 20 ng/µL prior to library preparation using dilution with nuclease-free water or evaporation.

WGS libraries were prepared using a modified version of the Illumina Nextera protocol (Karasov et al. 2018) with additional minor modifications as described below for use with vertebrates. In brief, 100 ng of extracted DNA from each individual was incubated for 10 min at 55 °C with Tn5 transposase to generate DNA fragments of variable size distribution that contained partial Illumina adapter sequences. Tagmentation products were amplified using the following PCR master mix solution as calculated for a single reaction: 8.875 of μL molecular-grade nuclease-free H_2_O, 2.500 μL of 5× Q5 Reaction Buffer (B9027S; New England Biolabs, Ipswich, Massachusetts), 0.125 μL of 5× Q5 High-Fidelty Polymerase (M0491L, New England Biolabs, Ipswich, Massachusetts), 0.500 μL of 10 mM dNTPs, 1.5 μL each of 5 mM custom oligonucleotides that included unique dual indexes and Illumina P7/P5 adapters, and 10 μL of tagmentation product for a total reaction volume of 25 μL. PCR thermocycler conditions were as follows: 72 °C for 10 min; 98 °C for 30 s; 12 cycles of 98 °C for 15 s; 65 °C for 20 s; and 72 °C for 3 min, with a final extension of 72 °C for 3 min.

Individual PCR products were validated via gel electrophoresis and were repeated for individuals that lacked bands or lacked DNA fragments of appropriate size (between 300 and 600 bp). Five-μL aliquots of each individual library were pooled, and size selection was performed using gel electrophoresis with band excision consisting of 5 total replicates of the initial library pool. DNA fragments of ~300 to 600 bp were excised for each replicate and purified using GeneJET Gel Extraction Kit (K0691; Thermo Scientific, Waltham, Massachusetts). The resulting size-selected pooled library replicates were combined to generate the final library pool. The concentration and DNA fragment size distribution for the final library pool were quantified using the dsDNA High Sensitivity Qubit Assay Kit (Q32854; Invitrogen, Waltham, Massachusetts) and TapeStation Agilent High Sensitivity D1000 ScreenTape Assay (5067; Agilent Technologies, Santa Clara, California). DNA sequencing of the final library pool was performed commercially (Novogene, Sacramento, California) using the NovaSeq X Plus 2 × 150 bp platform including 1% PhiX for quality control and targeting 3 × read coverage per individual haploid genome.

### Genome scaffolding.

Though quite complete, the currently available *S. niger* reference genome is highly fragmented (32,830 scaffolds; 183.8 kb N50; Kang et al. 2021). This fragmentation confounds variant calling, as it is unclear which sequences originate from the sex chromosomes and thus vary in ploidy based on the sex of the sequenced individual. To resolve this issue, we leveraged alignments against the chromosome-level *S. carolinensis* mSciCar1 genome (Blaxter et al. 2020)—the closest relative (Kumar et al. 2022) for which such a reference was available—to scaffold the vast majority of the *S. niger* assembly into pseudochromosomes for variant calling. For this approach, the *S. niger* genome was first processed with MitoHiFi v.1.4.1, and all identified mitochondrial sequences were removed. The remaining sequences were then aligned against the *S. carolinensis* mSciCar1 reference genome with RagTag v.2.1.0 (Alonge et al. 2022), requiring an alignment length of at least 37.5 kb (this value was chosen based on the N90 of the *S. niger* assembly). Scaffolds and contigs that did not reach this alignment threshold were retained as unplaced sequences. The pseudochromosomes resulting from this approach, which encompassed 80.5% of the *S. niger* assembly, were named based on their corresponding sequence in the *S. carolinensis* reference. For the generation of the mitochondrial genome, all identified candidate mitochondrial sequences were processed by MitoHiFi to build a consensus reference.

### Read quality control and mapping.

Sequencing adapters and low-quality base pairs for both species were trimmed using the default settings of Trim Galore! v.0.6.10 (Krueger et al. 2023), a wrapper for Cutadapt v.4.4 (Martin 2011) and FastQC v.0.12.1 (Andrew 2010). Following processing, reads were aligned using the default settings of BWA mem v.0.7.17 (Li 2013) to the pseudochromosome reference assembly. Aligned reads were coordinate-sorted using SAMtools v.1.17 (Li et al. 2009), duplicate reads were marked with Picard v.3.0.0 (Broad Institute 2019), and indels were left-aligned with GATK v.4.4.0.0 (Van der Auwera et al. 2020) to generate the BAM files used for variant calling.

### Variant calling and filtering.

Variants were jointly called across the 19 pseudoautosomes and mitochondrion using the mpileup and call commands of BCFtools v.1.16 (Danecek et al. 2021). Autosomal calls with a genotype quality less than 13 (equivalent to a genotyping confidence of 95%) and mitochondrial calls with a genotype quality less than 30 (equivalent to a genotyping confidence of 99.99%) were marked as uncalled (Kobayashi et al. 2017; Liu et al. 2023), indels and nonbiallelic single nucleotide polymorphism (SNPs) were removed, positions with more than 4 individuals missing data were filtered out (Guo et al. 2021; Magbanua et al. 2023), and all sites were required to have the minor allele present in at least 2 individuals (O’Leary et al. 2018). Autosomes were called using a ploidy value of 2, while mitochondrial calls were generated using a ploidy value of 1.

### Mitochondrial haplotype analysis.

The whole-mitochondrial genome variant call file (VCF) was filtered to only SNPs that had genotype calls in all 32 individuals using VCFtools v.0.1.16. The filtered mitochondrial VCF was converted into FASTA format using vcf2phylip v.2.0 (Ortiz 2019). The resulting FASTA file was imported into R using ape v.5.7-1 (Paradis and Schliep 2019) and converted into a multiple sequence alignment with Clustal v.2.1 (Higgins and Sharp 1988). Mitochondrial haplotypes were identified and plotted as a haplotype network with pegas v.1.3 (Paradis 2010).

### Principal component and identity-by-state analysis.

Variants for the autosomes were analyzed with “SNPRelate” v.1.36.0 (Zheng et al. 2012) running in an R v.4.3.2 (R Core Team 2021) environment. Autosomal SNPs were LD pruned using the following SNPRelate settings—method = composite; slide.max.bp = 2,000,000; ld.threshld = 0.1; start.pos = random. Principal component analysis (PCA) information for each data set was calculated with the snpgdsPCA function, while the snpgdsIBS function was used for identity-by-state (IBS) analysis. Clusters for the IBS trees were determined using 5,000 permutations and a *Z*-score threshold of 2.5.

### Relative heterozygosity analysis.

Although ~2× genome coverage is sufficient for the identification of polymorphic sites, when coupled with a small sample size, it can introduce biases in calls of allelic identity (Fumagalli 2013). Therefore, considering these limitations, we only compared relative rates of heterozygosity across individuals and did not calculate per site heterozygosity rates. We quantified the number of genome-wide heterozygous sites for each individual using a filtered data set of SNPs with a minimum site depth of 14 reads with PLINK v.2.00a2.3 (Chang et al. 2015). Relative heterozygosity was calculated for each individual by dividing individual heterozygosity rates (number of heterozygous sites divided by the total number of sites called for that individual) by the median heterozygosity rate across all individuals.

### iNaturalist data curation.

iNaturalist (iNaturallist Community 2024) observations were used for the visualization of fox squirrel and native tree squirrel sightings in California and Utah. Search criteria included the genus and species name for the Eastern Fox Squirrel and American Red Squirrel (*Tamiascirus hudsonicus*) in Utah, and the Eastern Fox Squirrel and Western Gray Squirrel (*Sciurus griseus*) in California. We restricted observations to only those that met the research grade standard for iNaturalist, which includes an observation date, georeference, photo, and a community-agreed species identification. As the fox squirrel and the native tree squirrel from each state are morphologically distinct, the research grade observations likely have a low frequency of incorrect species identifications. The observations were used to generate maps of sighting locations for each species in both California and Utah.

## Results

### Read coverage and variant identification.

After accounting for read trimming and PCR duplication, estimated genome coverage for the *S. niger* samples ranged from 0.93 to 10.12× (mean 3.04 ± 2.02 × SD) based on a genome size of 2.99 Gb (Kang et al. 2021; Supplementary Data SD1). 215 variant positions reached our genotyping threshold for the mitochondrial genome, while 745,282 variant positions met our criteria for the autosomes. Individual missingness was 0.00% to 26.05% for the mitochondrion (1.48 ± 5.04%; Supplementary Data SD2) and 3.08% to 27.59% (7.83 ± 5.26%; Supplementary Data SD3) for the autosomes. After removing mitochondrial sites with any missing data, the mitochondrial data set was reduced to 136 SNPs (Supplementary Data SD4). Following LD pruning, the autosomal data set was reduced to 13,961 unlinked SNPs with individual missingness of 1.91% to 31.69% (8.28 ± 6.32%; Supplementary Data SD5). The autosomal variant data set used for heterozygosity estimates contained 100,877 SNPs.

### Population structure.

**Fig. 2.**
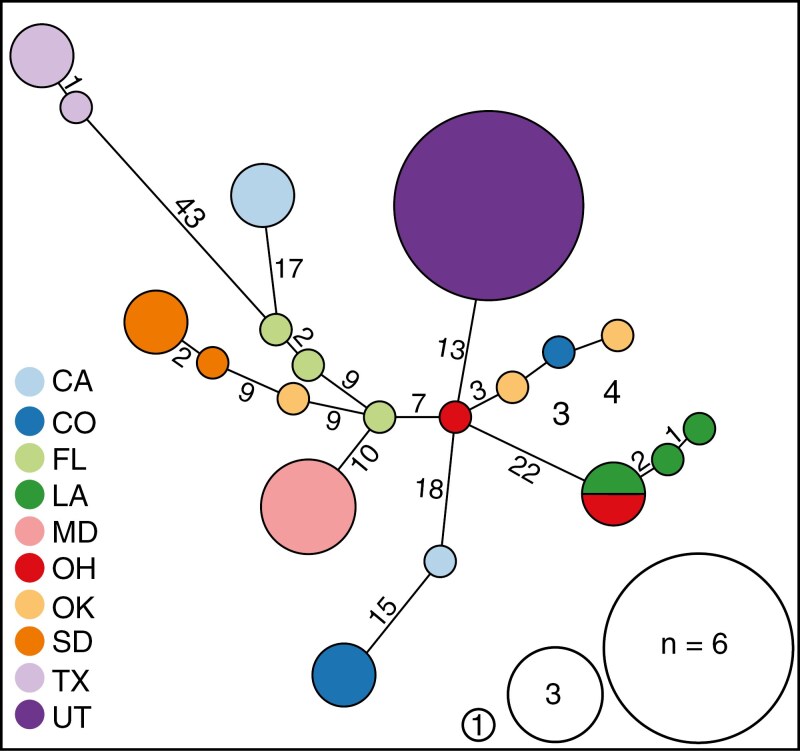
Haplotype network of *Sciurus niger* based on mitochondrial SNPs. Size of the circles indicates the number of individuals that share a haplotype. Color of the circles represents the state in which that haplotype is present. Numbers represent the number of SNPs separating each haplotype.

**Fig. 3.**
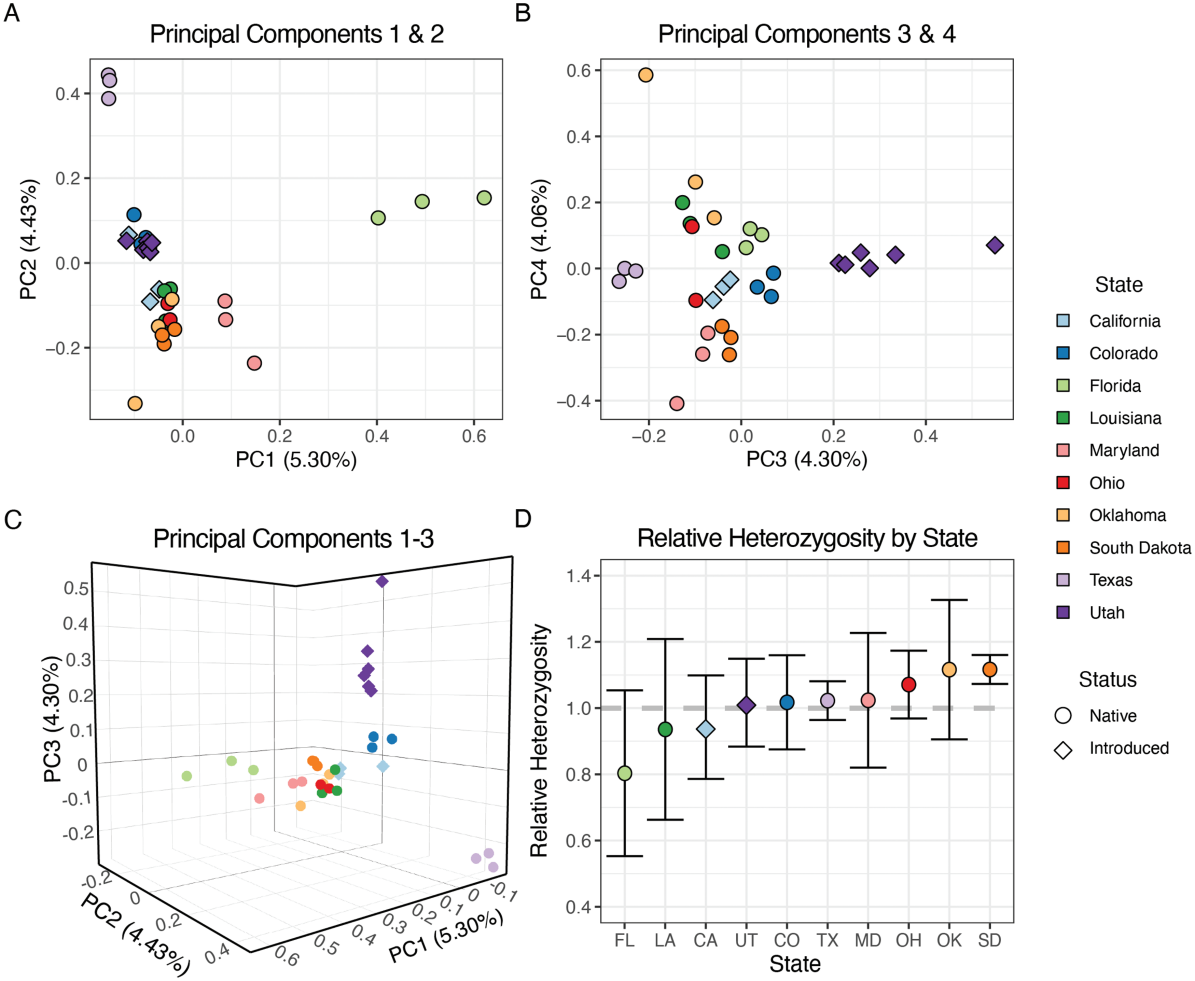
A visualization of population structure and genetic diversity of *Sciurus niger* based on 13,961 autosomal SNPs. Color represents the state individuals were sampled from and shape represents whether that individual is from an introduced or native population. (A) Principal component plot showing PC1 on the *x* axis and PC2 on the *y* axis. (B) Principal component plot showing PC3 on the *x* axis and PC4 on the *y* axis. (C) Three-dimensional principal component plot showing PC1 on the *x* axis, PC2 on the *z* axis, and PC3 on the *y* axis. (D) Mean relative heterozygosity for each population in our data set with error bars showing ±1 SD.

The mitochondrial haplotype network revealed 20 unique haplotypes across the introduced and native ranges of *S. niger* (Fig. 2); however, most individuals sampled from the same locality clustered closely together. We found that all individuals from Utah (*n* = 6) clustered most closely with a haplotype found in Ohio. For the California individuals, there were 2 haplotypes that were most similar to haplotypes present in Florida, while the third California haplotype was most similar to a haplotype found in Colorado (Fig. 2). PCA using the autosomal SNP data set revealed that Florida, Texas, and Maryland fox squirrels are genetically distinct from other populations in our data set (Fig. 3A). Similar to the mitochondrial analysis, we found that individuals were most similar to those sampled from the same geographic source (Fig. 3B). In this analysis, individuals from the introduced Utah population were most similar to those sampled in Colorado, 2 individuals from the introduced California population were most similar to those sampled in Louisiana, and the third individual from the introduced California population was most similar to those sampled in Colorado (Fig. 3A). We generated a 3-dimensional PCA plot to allow for visualization of the first 3 principal components (Fig. 3C). We compared the relative autosomal heterozygosity between individuals sampled from each geographic source to identify patterns of genetic diversity across both native and introduced sites (Fig. 3D). We found similar levels of relative heterozygosity across their range with 2 notable exceptions: Florida fox squirrels had low relative heterozygosity; and South Dakota had high relative heterozygosity when compared to other populations in our data set (Fig. 3D). IBS analysis was used to identify the degree of genetic similarity across sampling sites of *S. niger*. We found similar results to our PCA (Fig. 3A–C) with Florida, Texas, and Maryland being the most genetically dissimilar to other populations in our data set and individuals from the same sampling location generally clustering together, although overall genetic differentiation was low across all sampling localities (Fig. 5).

### iNaturalist

iNaturalist observations of native and introduced tree squirrels in California and Utah reveal similar patterns in each state. These observations indicate that *S. niger* is highly successful in urban environments, but this species appears to have a limited ability to colonize natural landscapes in either California or Utah to a significant degree (Fig. 5A and C). Native squirrels in each state successfully inhabit both urban and natural habitats (Fig. 5B and D). It should be noted that observations of native and introduced squirrels in both California and Utah are highest in urban environments, likely due to observation bias.

## Discussion

This study is the first to interrogate phylogeographic structure across the native and introduced ranges of a widespread tree squirrel in North America, *S. niger*, using WGS. We identified cryptic phylogeographic patterns in this species with few SNPs differentiating geographically distant populations. Additionally, the mitochondrial and autosomal analyses suggest that there have been multiple introductions into California, whereas Utah’s introduced population is likely the result of a single introduction event from a neighboring state. The minimal patterns of phylogeographic structure detected in this species are consistent with the recent range and population expansion this species has experienced since the end of the Last Glacial Maximum. We discuss these points in further detail below.

**Fig. 4.**
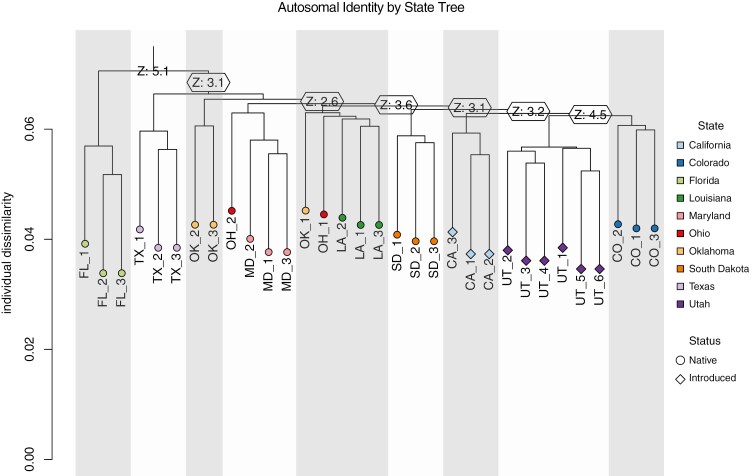
IBS tree showing relatedness between *Sciurus niger* individuals based on 13,961 autosomal SNPs. Color represents the state that individual was sampled from, and shape represents whether that individual was from an introduced or native population. Shaded/unshaded regions represent major population groupings based on *Z*-score cutoffs. Shorter branch lengths represent higher relatedness between individuals.

Despite overall limited genetic differentiation among sampling locations, fox squirrel populations in Florida and Texas were genetically distinct from other populations in North America. In some analyses, the Maryland population was also distinct from other populations in our data set (Figs. 2 and 3); however, this population shares a high degree of relatedness with individuals from Ohio and Oklahoma; thus, Maryland cannot be considered a genetically distinct subpopulation (Fig. 4). Furthermore, individuals in our data set tended to cluster together by sampling location, with the exception of the Oklahoma and California populations. Taken together, these findings reveal evidence of cryptic phylogeographic structure in this species. The phylogeographic pattern that we observed in *S. niger* is distinct from common phylogeographic patterns found in unglaciated North America (Soltis et al. 2006); however, a similar pattern is present in the flying squirrel (*Glaucomys volans*), which has Florida subpopulations that are genetically distinct from populations in the rest of North America and minimal genetic differentiation between populations (Petersen and Stewart 2006; Soltis et al. 2006). The similarities in phylogeographic structure between these 2 obligate tree squirrel species suggest that there may have been a single glacial refugium inhabited by tree squirrels during periods of recent glaciation, potentially located approximately to or within Florida.

This pattern of minimal genetic differentiation and similar levels of relative heterozygosity across the range of *S. niger* is consistent with rapid range and population expansion in the last 12 to 20 ka (Moncrief et al. 2012). The detection of phylogeographic structure, albeit minimal, in *S. niger* contradicts findings of past studies that used partial gene sequencing of the mitochondrial D-Loop and cytochrome *b* genes, which found no evidence for patterns of phylogeographic structure across either native or introduced sites (Moncrief et al. 2010, 2012; Claytor et al. 2015). However, the ability of this study to detect cryptic patterns of population structure is likely due to the increased resolution provided by WGS compared to the marker-gene sequencing approaches used previously.

We found 1 mitochondrial haplotype shared by all individuals sampled in Utah, and 2 haplotypes present in California. These results suggest that Utah’s population of *S. niger* likely originated from a single introduction event, while California’s population of *S. niger* originated from multiple introduction events. This finding is consistent with historical records of introductions of *S. niger* into California that catalog multiple intentional introduction events into California from 1901 to 1985 (Claytor et al. 2015). The presence of mito-nuclear discordance in California fox squirrels was further evidence of multiple fox squirrel introductions into California (Fig. 4; Supplementary Data SD6). In our analyses, California individuals shared mitochondrial haplotype structure most similar to either individuals sampled from Colorado or Florida, indicating these states may be the geographic sources of these introductions. Due to the unique mitochondrial haplotype present in Utah’s introduced fox squirrel population, the geographic source of this introduction remains unknown. However, based on genetic analysis from autosomal SNPs, Utah fox squirrels are most similar to fox squirrels in Colorado. Given this result combined with geographic adjacency, fox squirrels from Colorado or another neighboring state seem a likely source of those in Utah. Additionally, there is no record of an intentional introduction of *S. niger* into Utah, so Utah’s fox squirrel population may have been established by their unintentional transport through commercial shipping between states. It is worth mentioning that Utah’s fox squirrel population may have been introduced from another nonnative western population; however, this genetic signature would likely not be detectable given the recency of all western introductions.

**Fig. 5.**
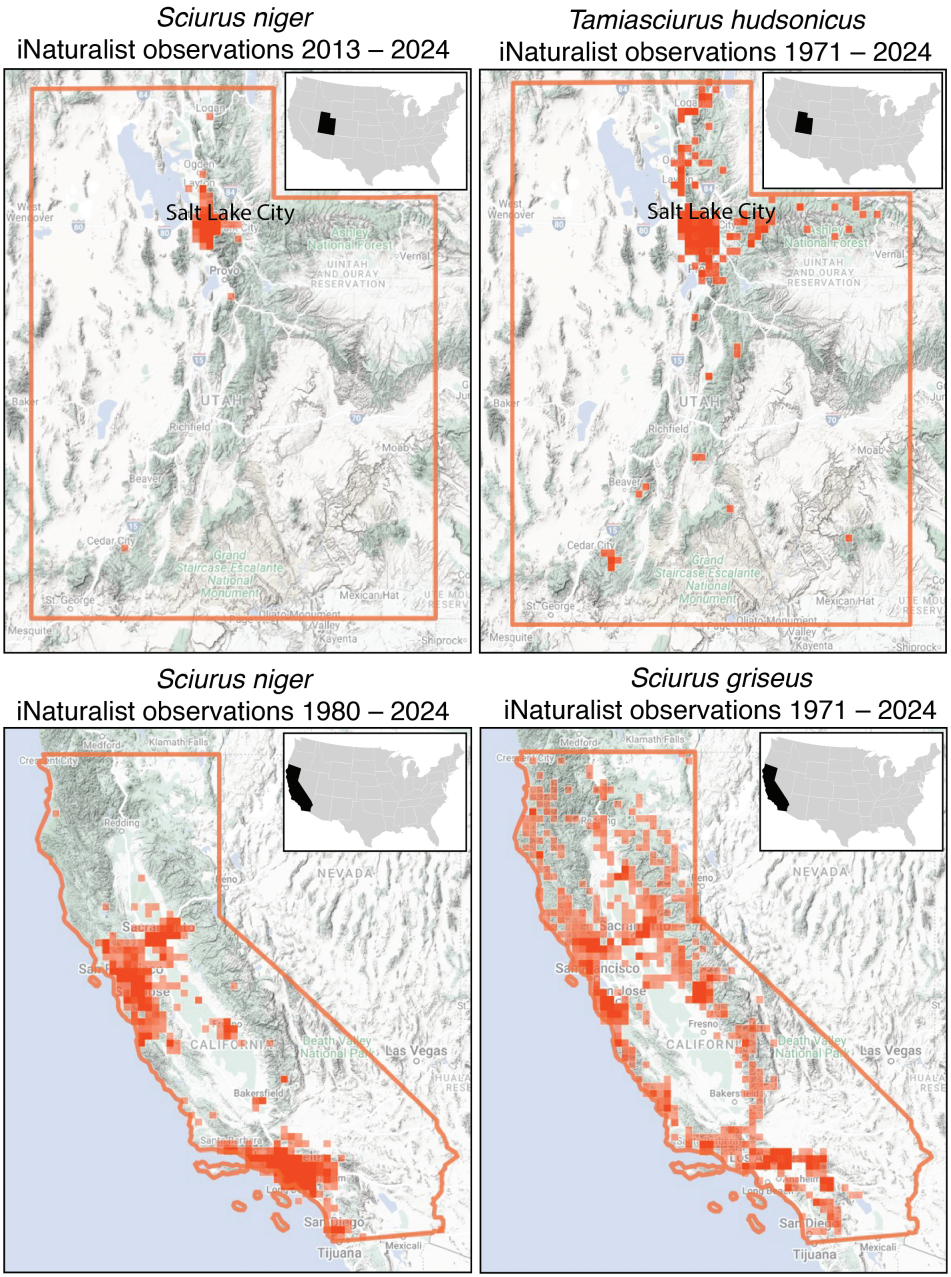
Population densities and range data of native and introduced tree squirrels in California and Utah based on iNaturalist observation data. Darker shading represents a higher density of observations in that area. (A) Observations of introduced fox squirrels (*Sciurus niger*) in Utah from 2013 to 2024. (B) Observations of native American red squirrels (*Tamiasciurus hudsonicus*) in Utah from 1971 to 2024. (C) Observations of introduced fox squirrels in California from 1980 to 2024. (D) Observations of native western gray squirrels (*Sciurus griseus*) in California from 1971 to 2024.

Native tree squirrels in California and Utah dominate natural environments in each state (Fig. 5B and D), while fox squirrels appear to be somewhat confined to urban areas (Fig. 5A and C). Since their initial sighting in the Salt Lake Valley in 2011, fox squirrels in Utah have continuously spread to urban centers throughout the state, but they have rarely been sighted in natural environments (Supplementary Data SD7). These results indicate that introduced fox squirrels may not represent a major threat to native tree squirrels in natural areas of western North America if they are unable to successfully colonize natural forest areas adjacent to urban centers. Furthermore, a study conducted in California found little evidence for interspecific competition in urban and suburban environments where *S. niger* and *S. griseus* live in sympatry (Ortiz 2022). Similar studies will need to be conducted in Utah to characterize potential interspecific interactions between native American red squirrels (*T. hudsonicus*) and *S. niger*. It is important to recognize that Utah’s fox squirrel introduction is very recent, and we have insufficient data to rule out their potential range expansion into more natural landscapes in the coming decades.

Historically, population genetics studies have relied on the use of microsatellites or marker genes (Sunnucks 2000; Yeap et al. 2011; Abdul-Muneer 2014; Sato et al. 2023), but continued advancements in next-generation sequencing have made WGS feasible for such studies in nonmodel species. The main advantage of WGS over other sequencing technologies is the timely sequencing of thousands or millions of genomic loci for a relatively low cost. In recent years, several WGS methods have been developed for use in population genomics, including restriction-site associated DNA sequencing (RAD-seq), POOL-seq, and low-pass WGS (lpWGS; Fuentes-Pardo and Ruzzante 2017). For our study, we used lpWGS, which has limitations and benefits over other WGS methods. This method is cost-effective and allows for population-level screening of the entire genome without sacrificing information on the individual level; however, low sequencing depth results in uncertainty for accurately calling variants or genotypes due to difficulty discriminating between real variants and sequencing errors (Fuentes-Pardo and Ruzzante 2017; Lou et al. 2021; Deng et al. 2022). The uncertainty caused by low sequencing depth forced us to use relative heterozygosity in our analyses as opposed to other measures of genetic diversity, and without true measures of heterozygosity, we were unable to perform admixture analyses or calculate effective population size. Another issue that we encountered was contiguity issues in the publicly available reference genome (Kang et al. 2021), even after manual improvements. Nonetheless, WGS has advantages over traditional marker gene analysis. For example, it generates much larger data sets that allow for the detection of minimal phylogeographic structure over a species range.

Although we were able to partially resolve the phylogeography of this species, a more expansive data set including more sampling localities and higher sequencing depth is needed to confidently pinpoint the exact geographic source of these introduction events and resolve phylogeographic structure across the full range of this species. Despite these limitations, this study improves our understanding of the genetic structure and connectivity of one of the most widespread rodent species in North America and will assist with managing the spread of fox squirrels to other localities in the western United States. For example, these data could be used to infer dispersal patterns of *S. niger* throughout the western United States, which has important implications for managing this species and preventing future invasions. Additionally, we generated publicly available genomic resources that lay the groundwork for future studies on *S. niger*, such as identifying genomic loci under selection in introduced populations, preventing future introductions through the identification of populations “preadapted” for invasion, and elucidating the geographic sources of future introductions and introduced western populations not captured in this study. As humans continue to alter landscapes and spatial distributions through climate change and globalization, similar studies will be increasingly vital to monitor the genetic structure and connectivity across the range of a species.

## Supplementary data

Supplementary data are available at *Journal of Mammalogy* online.

**Supplementary Data SD1.** Coverage metrics for the *S. niger* samples.

**Supplementary Data SD2.** Unpruned mitochondrial SNP metrics for *S. niger* individuals.

**Supplementary Data SD3.** Unpruned autosomal SNP metrics for *S. niger* individuals.

**Supplementary Data SD4.** Mitochondrial SNP metrics following the removal of sites with missing data for all *S. niger* individuals.

**Supplementary Data SD5.** Autosomal SNP metrics following LD pruning for all *S. niger* individuals.

**Supplementary Data SD6.** Identity-by-state tree showing relatedness between *Sciurus niger* individuals based on 136 mitochondrial SNPs in our data set. Color represents the state that individual was sampled from, and shape represents whether that individual originated in an introduced or native population. Shaded/unshaded regions represent major population groupings based on *Z*-score cutoffs. Shorter branch lengths represent higher relatedness between individuals.

**Supplementary Data SD7.** Figure cataloging the spread of *S. niger* throughout the state of Utah from 2013 to 2024 based on research grade iNaturalist observations. Each data point represents 1 fox squirrel sighting.

gyae133_suppl_Supplementary_Data_SD1

gyae133_suppl_Supplementary_Data_SD2

gyae133_suppl_Supplementary_Data_SD3

gyae133_suppl_Supplementary_Data_SD4

gyae133_suppl_Supplementary_Data_SD5

gyae133_suppl_Supplementary_Data_SD6

gyae133_suppl_Supplementary_Data_SD7

## Data Availability

All reads generated for this project have been deposited at the Sequence Read Archive under BioProject PRJNA1095500. The scaffolded chromosomal genome produced for this project, as well as all accompanying VCFs have been deposited at the Open Science Foundation and are available at https://osf.io/xwzuk/.
