# Correction to: The Eastern Fox Squirrel (Sciurus niger) exhibits minimal patterns of phylogeography across native and introduced sites

**DOI:** 10.1093/jmammal/gyaf005

**Published:** 2025-02-13

**Keywords:** ardilla zorro oriental, especies introducidas, genética de poblaciones, roedor, secuenciación del genoma

## Abstract

Especies introducidas son una de las principales causes de la disminución mundial de la biodiversidad y resultan en pérdidas de miles de millones de dólares para la bioeconomía mundial. La genética de poblaciones es un instrumento útil para el manejo de estas especies, cada vez más comunes debido a la globalización y el cambio climático. La ardilla zorro oriental (*Sciurus niger*) es una especie invasora exitosa que se introdujo en muchos estados del oeste de América del Norte a lo largo del siglo XX. Utilizamos secuenciación de baja cobertura del genoma para evaluar la estructura filogeográfica de la ardilla zorro oriental a través sitios nativos e introducidos, para identificar el número de introducciones en los estados de California y Utah, y para determinar orígenes geográficos de estas introducciones. Encontramos una señal mínima de estructura filogeográfica, consistente con la reciente expansión de rango y población de esta especie desde el ultimo Máximo Glacial. Además, encontramos múltiples haplotipos mitocondriales en California y solo uno haplotipo en Utah. Esto sugiere que en California, la ardilla zorro oriental se originó de múltiples eventos de introducción, mientras en Utah se originó a partir de un solo evento. Los recursos genómicos de este estudio serán útiles para futuros proyectos de conservación de esta especie y ayudarán con la gestión de sus introducciones en el oeste de América del Norte.

This is a correction to:

Noah Armstrong, Dylan M Klure, Robert Greenhalgh, Tess E Stapleton, M Denise Dearing, The Eastern Fox Squirrel (Sciurus niger) exhibits minimal patterns of phylogeography across native and introduced sites, Journal of Mammalogy, 2024;, gyae133, https://doi.org/10.1093/jmammal/gyae133

The following changes have been made to the originally published manuscript: the foreign language translation for the title and abstract have been added to the article.

