# Supplementary material for: The Eastern Fox Squirrel (Sciurus niger) exhibits minimal patterns of phylogeography across native and introduced sites
Source: J Mammal. 2024 Nov 15;106(2):394–404. doi: 10.1093/jmammal/gyae133 (PMC11933279; doi:10.1093/jmammal/gyae133)
Supplement: gyae133_suppl_Supplementary_Data_SD1 [file gyae133_suppl_supplementary_data_sd1.docx]

**Table S1.** Coverage metrics for the *S. niger* samples

| Sample | Read bp (forward) | Read bp (reverse) | Read bp (total) | Coverage (all reads) | Duplication % | Coverage (effective) |
| --- | --- | --- | --- | --- | --- | --- |
| CA_01 | 4,157,344,002 | 4,126,765,773 | 8,284,109,775 | 2.77 | 11.86% | 2.44 |
| CA_02 | 2,473,027,398 | 2,458,680,300 | 4,931,707,698 | 1.65 | 10.76% | 1.47 |
| CA_03 | 4,876,851,041 | 4,844,979,026 | 9,721,830,067 | 3.25 | 11.62% | 2.87 |
| CO_01 | 2,851,591,870 | 2,834,873,032 | 5,686,464,902 | 1.90 | 10.68% | 1.70 |
| CO_02 | 5,624,032,665 | 5,586,800,731 | 11,210,833,396 | 3.75 | 11.58% | 3.32 |
| CO_03 | 3,575,436,180 | 3,556,430,534 | 7,131,866,714 | 2.39 | 10.30% | 2.14 |
| FL_01 | 4,428,765,937 | 4,408,474,587 | 8,837,240,524 | 2.96 | 11.19% | 2.62 |
| FL_02 | 5,192,562,123 | 5,214,425,926 | 10,406,988,049 | 3.48 | 8.33% | 3.19 |
| FL_03 | 2,074,481,988 | 2,065,630,061 | 4,140,112,049 | 1.38 | 6.74% | 1.29 |
| LA_01 | 1,499,160,033 | 1,491,849,139 | 2,991,009,172 | 1.00 | 6.67% | 0.93 |
| LA_02 | 3,833,049,919 | 3,823,815,680 | 7,656,865,599 | 2.56 | 12.23% | 2.25 |
| LA_03 | 5,776,415,017 | 5,788,082,843 | 11,564,497,860 | 3.87 | 11.17% | 3.44 |
| MD_01 | 14,045,355,377 | 13,993,378,235 | 28,038,733,612 | 9.38 | 11.36% | 8.31 |
| MD_02 | 3,504,764,326 | 3,479,687,432 | 6,984,451,758 | 2.34 | 10.83% | 2.08 |
| MD_03 | 2,832,692,429 | 2,821,182,778 | 5,653,875,207 | 1.89 | 8.95% | 1.72 |
| OH_01 | 3,334,924,039 | 3,320,080,212 | 6,655,004,251 | 2.23 | 7.96% | 2.05 |
| OH_02 | 5,992,484,731 | 5,981,538,634 | 11,974,023,365 | 4.00 | 7.17% | 3.72 |
| OK_01 | 3,536,137,864 | 3,539,827,589 | 7,075,965,453 | 2.37 | 8.22% | 2.17 |
| OK_02 | 3,920,827,888 | 3,900,511,513 | 7,821,339,401 | 2.62 | 7.27% | 2.43 |
| OK_03 | 16,864,332,794 | 16,809,323,044 | 33,673,655,838 | 11.26 | 10.11% | 10.12 |
| SD_01 | 6,495,445,860 | 6,409,422,795 | 12,904,868,655 | 4.32 | 6.39% | 4.04 |
| SD_02 | 6,624,439,272 | 6,600,844,442 | 13,225,283,714 | 4.42 | 6.66% | 4.13 |
| SD_03 | 4,859,700,614 | 4,855,810,193 | 9,715,510,807 | 3.25 | 8.26% | 2.98 |
| TX_01 | 5,338,949,952 | 5,313,043,048 | 10,651,993,000 | 3.56 | 11.76% | 3.14 |
| TX_02 | 3,848,755,344 | 3,831,100,585 | 7,679,855,929 | 2.57 | 11.25% | 2.28 |
| TX_03 | 4,875,164,247 | 4,855,185,148 | 9,730,349,395 | 3.25 | 10.77% | 2.90 |
| UT_01 | 2,905,723,992 | 2,892,567,225 | 5,798,291,217 | 1.94 | 10.95% | 1.73 |
| UT_02 | 3,386,085,593 | 3,377,440,776 | 6,763,526,369 | 2.26 | 9.95% | 2.04 |
| UT_03 | 3,914,220,620 | 3,902,640,858 | 7,816,861,478 | 2.61 | 9.03% | 2.38 |
| UT_04 | 3,654,928,792 | 3,634,667,702 | 7,289,596,494 | 2.44 | 8.60% | 2.23 |
| UT_05 | 12,683,465,953 | 12,631,589,758 | 25,315,055,711 | 8.47 | 10.25% | 7.60 |
| UT_06 | 2,905,791,207 | 2,884,099,545 | 5,789,890,752 | 1.94 | 11.52% | 1.71 |
