# Supplementary material for: The Eastern Fox Squirrel (Sciurus niger) exhibits minimal patterns of phylogeography across native and introduced sites
Source: J Mammal. 2024 Nov 15;106(2):394–404. doi: 10.1093/jmammal/gyae133 (PMC11933279; doi:10.1093/jmammal/gyae133)
Supplement: gyae133_suppl_Supplementary_Data_SD2 [file gyae133_suppl_supplementary_data_sd2.docx]

**Table S2.** Unpruned mitochondrial SNP metrics for *S. niger* individuals

| Sample | Missing sites | Called sites | Missing % | Called % |
| --- | --- | --- | --- | --- |
| CA_01 | 0 | 215 | 0.00% | 100.00% |
| CA_02 | 0 | 215 | 0.00% | 100.00% |
| CA_03 | 0 | 215 | 0.00% | 100.00% |
| CO_01 | 0 | 215 | 0.00% | 100.00% |
| CO_02 | 0 | 215 | 0.00% | 100.00% |
| CO_03 | 0 | 215 | 0.00% | 100.00% |
| FL_01 | 0 | 215 | 0.00% | 100.00% |
| FL_02 | 56 | 159 | 26.05% | 73.95% |
| FL_03 | 3 | 212 | 1.40% | 98.60% |
| LA_01 | 0 | 215 | 0.00% | 100.00% |
| LA_02 | 2 | 213 | 0.93% | 99.07% |
| LA_03 | 0 | 215 | 0.00% | 100.00% |
| MD_01 | 1 | 214 | 0.47% | 99.53% |
| MD_02 | 0 | 215 | 0.00% | 100.00% |
| MD_03 | 0 | 215 | 0.00% | 100.00% |
| OH_01 | 0 | 215 | 0.00% | 100.00% |
| OH_02 | 28 | 187 | 13.02% | 86.98% |
| OK_01 | 0 | 215 | 0.00% | 100.00% |
| OK_02 | 0 | 215 | 0.00% | 100.00% |
| OK_03 | 2 | 213 | 0.93% | 99.07% |
| SD_01 | 0 | 215 | 0.00% | 100.00% |
| SD_02 | 1 | 214 | 0.47% | 99.53% |
| SD_03 | 0 | 215 | 0.00% | 100.00% |
| TX_01 | 0 | 215 | 0.00% | 100.00% |
| TX_02 | 0 | 215 | 0.00% | 100.00% |
| UT_01 | 0 | 215 | 0.00% | 100.00% |
| UT_02 | 1 | 214 | 0.47% | 99.53% |
| UT_03 | 1 | 214 | 0.47% | 99.53% |
| UT_04 | 4 | 211 | 1.86% | 98.14% |
| UT_05 | 0 | 215 | 0.00% | 100.00% |
| UT_06 | 3 | 212 | 1.40% | 98.60% |
