# Supplementary material for: The Eastern Fox Squirrel (Sciurus niger) exhibits minimal patterns of phylogeography across native and introduced sites
Source: J Mammal. 2024 Nov 15;106(2):394–404. doi: 10.1093/jmammal/gyae133 (PMC11933279; doi:10.1093/jmammal/gyae133)
Supplement: gyae133_suppl_Supplementary_Data_SD3 [file gyae133_suppl_supplementary_data_sd3.docx]

**Table S3:** Unpruned autosomal SNP metrics for *S. niger* individuals

| Sample | Missing Sites | Homozygous Sites | Heterozygous Sites | Missing % | Homozygous % | Heterozygous % |
| --- | --- | --- | --- | --- | --- | --- |
| CA_01 | 49,955 | 572,837 | 122,490 | 6.70% | 76.86% | 16.44% |
| CA_02 | 93,883 | 553,421 | 97,978 | 12.60% | 74.26% | 13.15% |
| CA_03 | 39,029 | 580,220 | 126,033 | 5.24% | 77.85% | 16.91% |
| CO_01 | 70,355 | 566,188 | 108,739 | 9.44% | 75.97% | 14.59% |
| CO_02 | 38,490 | 563,584 | 143,208 | 5.16% | 75.62% | 19.22% |
| CO_03 | 57,772 | 565,613 | 121,897 | 7.75% | 75.89% | 16.36% |
| FL_01 | 52,292 | 550,751 | 142,239 | 7.02% | 73.90% | 19.09% |
| FL_02 | 36,974 | 551,495 | 156,813 | 4.96% | 74.00% | 21.04% |
| FL_03 | 205,658 | 454,471 | 85,153 | 27.59% | 60.98% | 11.43% |
| LA_01 | 186,785 | 482,660 | 75,837 | 25.06% | 64.76% | 10.18% |
| LA_02 | 52,706 | 571,145 | 121,431 | 7.07% | 76.63% | 16.29% |
| LA_03 | 38,340 | 563,569 | 143,373 | 5.14% | 75.62% | 19.24% |
| MD_01 | 22,952 | 556,472 | 165,858 | 3.08% | 74.67% | 22.25% |
| MD_02 | 54,448 | 569,531 | 121,303 | 7.31% | 76.42% | 16.28% |
| MD_03 | 70,110 | 558,620 | 116,552 | 9.41% | 74.95% | 15.64% |
| OH_01 | 58,512 | 564,082 | 122,688 | 7.85% | 75.69% | 16.46% |
| OH_02 | 44,681 | 559,944 | 140,657 | 6.00% | 75.13% | 18.87% |
| OK_01 | 50,886 | 575,559 | 118,837 | 6.83% | 77.23% | 15.95% |
| OK_02 | 45,839 | 579,623 | 119,820 | 6.15% | 77.77% | 16.08% |
| OK_03 | 23,976 | 546,807 | 174,499 | 3.22% | 73.37% | 23.41% |
| SD_01 | 36,851 | 574,347 | 134,084 | 4.94% | 77.06% | 17.99% |
| SD_02 | 38,659 | 565,770 | 140,853 | 5.19% | 75.91% | 18.90% |
| SD_03 | 44,493 | 570,757 | 130,032 | 5.97% | 76.58% | 17.45% |
| TX_01 | 46,354 | 564,207 | 134,721 | 6.22% | 75.70% | 18.08% |
| TX_02 | 49,339 | 566,124 | 129,819 | 6.62% | 75.96% | 17.42% |
| TX_03 | 39,761 | 566,941 | 138,580 | 5.34% | 76.07% | 18.59% |
| UT_01 | 72,630 | 562,943 | 109,709 | 9.75% | 75.53% | 14.72% |
| UT_02 | 56,564 | 566,294 | 122,424 | 7.59% | 75.98% | 16.43% |
| UT_03 | 46,406 | 576,131 | 122,745 | 6.23% | 77.30% | 16.47% |
| UT_04 | 50,189 | 574,718 | 120,375 | 6.73% | 77.11% | 16.15% |
| UT_05 | 23,783 | 565,023 | 156,476 | 3.19% | 75.81% | 21.00% |
| UT_06 | 69,602 | 566,855 | 108,825 | 9.34% | 76.06% | 14.60% |
