# Supplementary material for: The Eastern Fox Squirrel (Sciurus niger) exhibits minimal patterns of phylogeography across native and introduced sites
Source: J Mammal. 2024 Nov 15;106(2):394–404. doi: 10.1093/jmammal/gyae133 (PMC11933279; doi:10.1093/jmammal/gyae133)
Supplement: gyae133_suppl_Supplementary_Data_SD5 [file gyae133_suppl_supplementary_data_sd5.docx]

**Table S5:** Autosomal SNP metrics following LD pruning for all *S. niger* individuals

| Sample | Missing Sites | Homozygous Sites | Heterozygous Sites | Missing % | Homozygous % | Heterozygous % |
| --- | --- | --- | --- | --- | --- | --- |
| CA_01 | 902 | 11,629 | 1,430 | 6.46% | 83.30% | 10.24% |
| CA_02 | 2,066 | 10,662 | 1,233 | 14.80% | 76.37% | 8.83% |
| CA_03 | 669 | 11,816 | 1,476 | 4.79% | 84.64% | 10.57% |
| CO_01 | 1,507 | 11,097 | 1,357 | 10.79% | 79.49% | 9.72% |
| CO_02 | 568 | 11,855 | 1,538 | 4.07% | 84.92% | 11.02% |
| CO_03 | 1,199 | 11,349 | 1,413 | 8.59% | 81.29% | 10.12% |
| FL_01 | 1,119 | 11,318 | 1,524 | 8.02% | 81.07% | 10.92% |
| FL_02 | 579 | 11,758 | 1,624 | 4.15% | 84.22% | 11.63% |
| FL_03 | 3,760 | 9,044 | 1,157 | 26.93% | 64.78% | 8.29% |
| LA_01 | 4,424 | 8,545 | 992 | 31.69% | 61.21% | 7.11% |
| LA_02 | 1,072 | 11,478 | 1,411 | 7.68% | 82.21% | 10.11% |
| LA_03 | 554 | 11,846 | 1,561 | 3.97% | 84.85% | 11.18% |
| MD_01 | 267 | 12,073 | 1,621 | 1.91% | 86.48% | 11.61% |
| MD_02 | 1,145 | 11,437 | 1,379 | 8.20% | 81.92% | 9.88% |
| MD_03 | 1,600 | 11,019 | 1,342 | 11.46% | 78.93% | 9.61% |
| OH_01 | 1,243 | 11,301 | 1,417 | 8.90% | 80.95% | 10.15% |
| OH_02 | 856 | 11,613 | 1,492 | 6.13% | 83.18% | 10.69% |
| OK_01 | 965 | 11,556 | 1,440 | 6.91% | 82.77% | 10.31% |
| OK_02 | 855 | 11,674 | 1,432 | 6.12% | 83.62% | 10.26% |
| OK_03 | 325 | 11,960 | 1,676 | 2.33% | 85.67% | 12.00% |
| SD_01 | 552 | 11,935 | 1,474 | 3.95% | 85.49% | 10.56% |
| SD_02 | 649 | 11,784 | 1,528 | 4.65% | 84.41% | 10.94% |
| SD_03 | 834 | 11,650 | 1,477 | 5.97% | 83.45% | 10.58% |
| TX_01 | 844 | 11,562 | 1,555 | 6.05% | 82.82% | 11.14% |
| TX_02 | 933 | 11,566 | 1,462 | 6.68% | 82.85% | 10.47% |
| TX_03 | 651 | 11,789 | 1,521 | 4.66% | 84.44% | 10.89% |
| UT_01 | 1,804 | 10,860 | 1,297 | 12.92% | 77.79% | 9.29% |
| UT_02 | 1,179 | 11,427 | 1,355 | 8.44% | 81.85% | 9.71% |
| UT_03 | 880 | 11,682 | 1,399 | 6.30% | 83.68% | 10.02% |
| UT_04 | 1,051 | 11,524 | 1,386 | 7.53% | 82.54% | 9.93% |
| UT_05 | 348 | 12,037 | 1,576 | 2.49% | 86.22% | 11.29% |
| UT_06 | 1,600 | 11,081 | 1,280 | 11.46% | 79.37% | 9.17% |
