# Supplementary figures and images for: The Eastern Fox Squirrel (Sciurus niger) exhibits minimal patterns of phylogeography across native and introduced sites
Source: J Mammal. 2024 Nov 15;106(2):394–404. doi: 10.1093/jmammal/gyae133 (PMC11933279; doi:10.1093/jmammal/gyae133)

# Mitochondrial Identity by State Tree

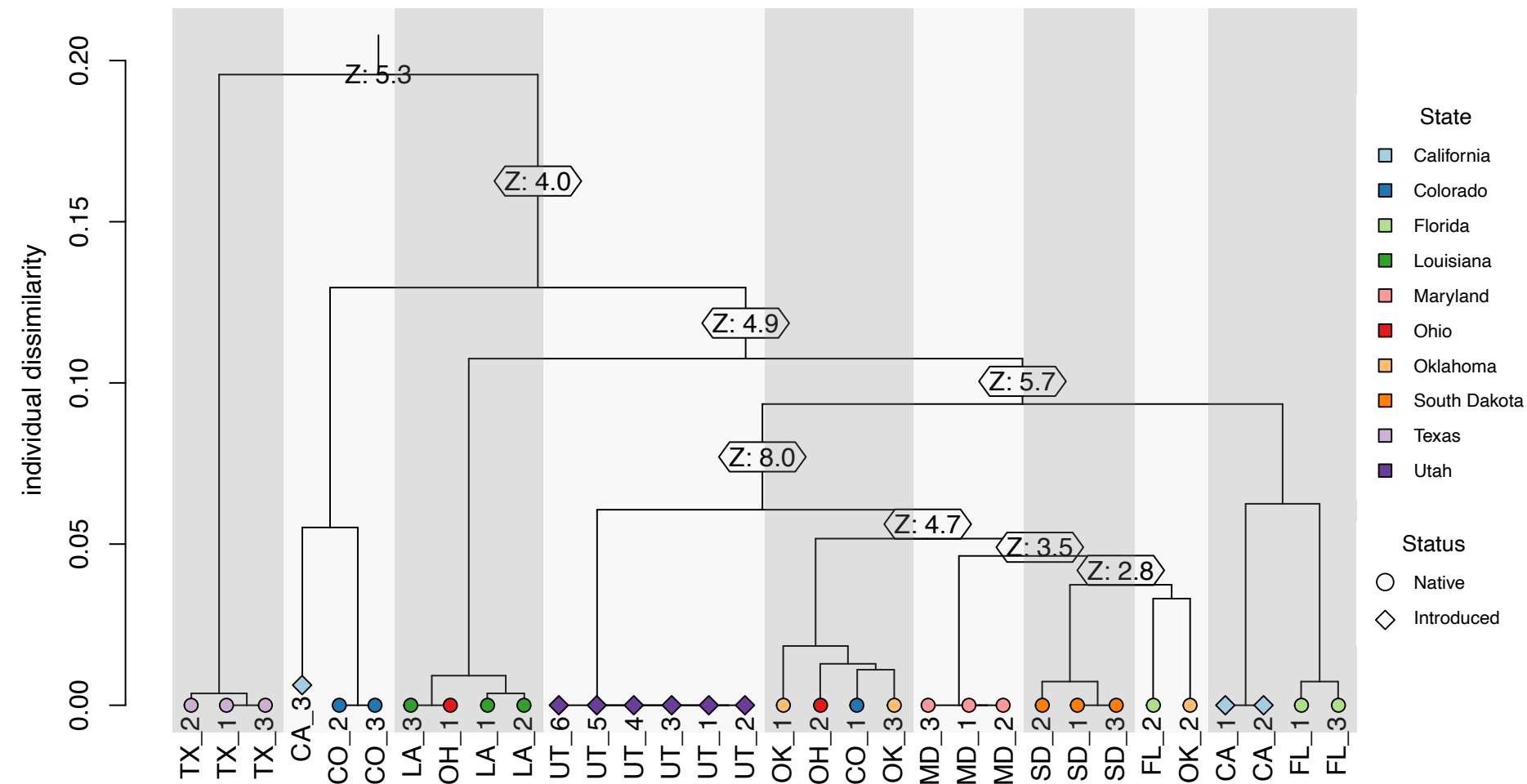

Supplement: gyae133_suppl_Supplementary_Data_SD6 [file gyae133_suppl_supplementary_data_sd6.pdf]

| 2013 | 2014 | 2015 | 2016 |
|------|------|------|------|
|------|------|------|------|

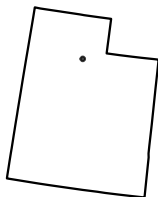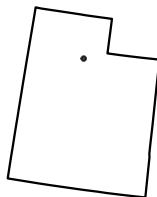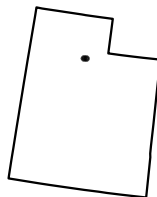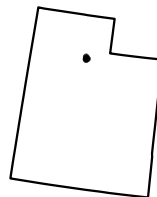

| 2017 | 2018 | 2019 | 2020 |
|------|------|------|------|
|------|------|------|------|

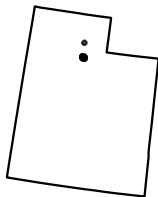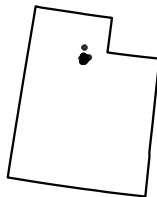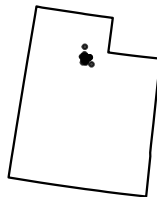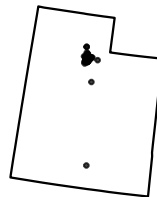

| 2021 | 2022 | 2023 | 2024 |
|------|------|------|------|
|------|------|------|------|

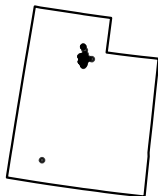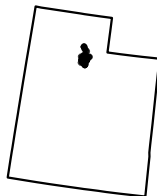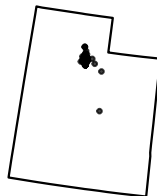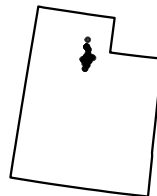

Supplement: gyae133_suppl_Supplementary_Data_SD7 [file gyae133_suppl_supplementary_data_sd7.pdf]
